# A multicenter cross-sectional survey of the knowledge, attitude, and behaviour of nurses regarding dysphagia after anterior cervical spine surgery: a prospective multicentre study

**DOI:** 10.1186/s12912-023-01690-2

**Published:** 2024-01-29

**Authors:** Chen Yu, Luo Chunmei, Song Caiping

**Affiliations:** Xinqiao Hospital, The Army Medical University, Chongqing, 400037 China

**Keywords:** Knowledge, Attitudes, Anterior cervical spine surgery, Dysphagia

## Abstract

**Background:**

The incidence of dysphagia after anterior cervical spine surgery is high, which directly affects the quality of life of patients after surgery. The knowledge, attitude, and behavior of nurses can affect the identification and management of patients after anterior cervical spine surgery. Therefore, we need a survey to understand the current status of nurses’ knowledge, attitude, and behavior towards dysphagia after anterior cervical spine surgery.

**Objective:**

To investigate the knowledge, attitude, and behaviour of orthopaedic nurses towards patients with dysphagia after anterior cervical spine surgery and provide recommendations for management and intervention.

**Method:**

An online cross-sectional survey was conducted between March and June 2023, which among 894 orthopaedic nurses from 36 tertiary hospitals in Chongqing using a questionnaire. The questionnaire included general information and knowledge, attitudes, and behaviours related to the management of dysphagia after anterior cervical surgery.

**Results:**

The orthopaedic nurses’ mean score for dysphagia-related knowledge was 16.1 ± 3.640 (out of a total score of 30). The orthopaedic nurses’ mean score for dysphagia-related attitude was 32.5 ± 4.623 (out of a total score of 40). The orthopaedic nurses’ mean score for dysphagia-related behaviour was 43.6 ± 11.513 (out of a total score of 60). The results of statistical analysis showed that the dysphagia after anterior cervical spine surgery -related knowledge scores differed significantly among the nurses according to, education level, and training (P < 0.05). Correlation analysis showed that there was a positive correlation between the knowledge, attitude, and behaviour scores of neurological nurses and swallowing disorders after anterior cervical surgery (P < 0.05).

**Conclusion:**

Targeted knowledge and skills training should be carried out to improve the rules and regulations for dysphagia after anterior cervical spine surgery. Multidisciplinary team cooperation is needed, and dietary service processes and management standards should be improved to improve the management ability of orthopaedic nurses for dysphagia after anterior cervical spine surgery.

**Supplementary Information:**

The online version contains supplementary material available at 10.1186/s12912-023-01690-2.

## Introduction

Anterior cervical spine surgery (ACSS) has become the preferred treatment for cervical spondylosis because it can directly and completely relieve spinal cord compression [1–2]. Dysphagia is the most common complication after ACSS, with an early incidence of 88.8% [3]. Dysphagia after surgery reduces the quality of life of patients, prolongs hospital stays, and increases treatment costs [4–5]. More serious complications, such as aspiration pneumonia, malnutrition and asphyxia, can also occur [6]. Orthopaedic nurses are the medical personnel who have the most direct contact with patients after cervical spine surgery and play a vital role in the multidisciplinary cooperative management of dysphagia after cervical spine surgery. For patients suspected of having swallowing problems, screening for dysphagia should be carried out. The screening is generally completed by nurses, and other professionals can also participate [7]. In addition, after identifying patients with dysphagia, careful management should be carried out to achieve the best outcome for patients. The status quo of nurses’ knowledge, attitudes and behaviours towards dysphagia directly affects their recognition and management of dysphagia in cervical surgery [8]. However, due to the lack of investigation and research in this area, it is necessary for the researchers to investigate the existing status of orthopaedic nurses’ knowledge, attitude and behaviours regarding dysphagia, understand them, provide a basis for managers and educators to carry out relevant training, and improve the quality of care to ensure patient safety.

## Materials and methods

### Study design and participants

An online cross-sectional survey was conducted between March and June 2023. We used an online questionnaire system called Question Star to collect the data (https://www.wjx.cn/login.aspx). This system is widely used in the academic community of China. The questionnaire link generated by Question Star was shared with eligible medical staff through social media platforms such as WeChat. To reduce the risk of duplicate submissions, IP address restriction technology was used.

Using a convenience sampling method, 894 nurses from orthopaedics departments of 17 hospitals in Chongqing from September to October 2019 were selected as the research subjects. The inclusion criteria for survey participants were as follows: (1) orthopaedic nurses; (2) nurses with working experience of 6 months or more. The exclusion criteria were as follows: personnel who were not currently working (on vacation, studying abroad, etc.). The survey subjects all signed informed consent and voluntarily participated in this study, which met the requirements of the Declaration of Helsinki.

### Data collection instruments

This study referred to the swallowing disorder knowledge questionnaire constructed by Rhoda [9], and based on the evidence summary conducted by the previous research group, combined with our previous clinical qualitative interviews [10], a preliminary draft of the questionnaire was drafted. After the initial draft was produced, six experts (one chief orthopaedic physician from a tertiary hospital, two deputy chief orthopaedic nurses, two deputy chief brain surgery nurses, and one deputy chief otolaryngologist) were invited to screen and modify each entry. Experts reviewed and modified the relevance and adaptability of each knowledge item, deleting a total of 6 knowledge items, adding 5 knowledge items, and modifying 3 knowledge items, ultimately forming a survey questionnaire with a total of 30 items.

The survey questionnaire consisted of four parts as follows: (1) General information. There were 18 items, including the hospital where the nurses worked, the level of the hospital, whether it was a teaching hospital, age, sex, initial education level, highest education level, professional title, nursing years, length of service in orthopaedics, position, number of times the nurses had received swallowing disorder-related training (lectures or online), and total duration of swallowing disorder-related training. (2) Orthopaedic nurses’ knowledge questionnaire on swallowing disorders after anterior cervical spine surgery. There were a total of 30 items in the questionnaire, all of which were multiple-choice questions. If the answer was correct, 1 point was given, and if the answer was incorrect or could not be judged, 0 points were given. The total score was 30 points. The higher the score, the better the knowledge mastery. (3) Poststroke swallowing disorder nursing belief questionnaire for orthopaedic nurses. There were 8 items in total, using the Likert 5-level scoring method. “Greatly agree” was 5 points, “agree” was 4 points, “neutral”” was 3 points, “disagree” was 2 points, and “greatly disagree” was 1 point, with a total score of 5–40 points. The higher the score, the better the nurse’s belief in nursing care for swallowing disorders after stroke. (4) Neurological nurses’ questionnaire on nursing behaviours for dysphagia after stroke. There were a total of 12 items, using the Likert 5-level scoring method. “always” was 5 points, “often” was 4 points, “sometimes” was 3 points, “rarely” was 2 points, and “never” was 1 point, with a total score of 0–60 points. The survey subjects were asked to answer based on their daily work situation. The higher the score, the better the nursing behaviour of nurses towards swallowing disorders after stroke. If the score was less than 50% of the total score (< 30 points), it was recorded as poor, 50% -<75% (30–45 points) was recorded as moderate, and 75 -100% (45–60 points) was recorded as good. The survey questionnaire was ultimately evaluated for content validity by 6 experts. The content validity was evaluated using a 4-point method, where 1 = irrelevant, 2 = somewhat relevant, 3 = relevant, and 4 = very relevant. The content validity index calculated using the average of all items was 0.93, indicating good content validity. The Cronbach’s α coefficient of the questionnaire was 0.897. Before distributing the survey questionnaire, 30 orthopaedic nurses were selected with a reasonable distribution of age and professional titles, and retests were conducted at 2-week intervals to assess the reliability of the retest. Pearson’s correlation coefficient was 0.827, indicating the high retest reliability of the questionnaire.

### Procedures

The questionnaire survey was conducted using Question Star, and the purpose and importance of the research were described in the questionnaire description. Completion of all items was mandatory, and those nurses who had missing responses could not submit the questionnaire. Each IP address could only be submitted once to ensure the reliability of the obtained data. There was one person in charge at each of the 36 hospitals who sent the questionnaire to eligible orthopaedic nurses in this hospital through WeChat. Nurses answered through WeChat on their mobile phones, submitted the questionnaihre, and researchers downloaded and collected the questionnaires from the platform. A total of 894 questionnaires were collected, with 894 valid questionnaires and a 100% effective response rate.

### Ethical considerations

Before starting the investigation, all participants were informed of the research purpose and provided informed consent forms (through online forms). All participants ensured the anonymity and confidentiality of their responses and data, as well as their right to withdraw from the study at any time. All study procedures were approved by the Medical Ethics Committee of Second Affiliated Hospital of Army Medical University, PLA (2022-430-01).

### Data analysis

All data were exported from Question Star to SPSS 21.0 statistical software for logical error checking, and statistical analysis was conducted after confirming that all questionnaires were valid. The measurement data with a normal distribution and uniform variance are expressed as the means ± standard deviations (x ± s). The comparison between two groups was performed by the t test, and the comparison between multiple groups was performed by analysis of variance. The counting data are represented by frequencies, percentages (%), and categorical variables are described as percentages and were compared using the chi-square test. Pearson correlation analysis was used to analyse the correlation between age and dysphagia nursing knowledge, attitude and behaviour scores and the correlation among nurses’ dysphagia knowledge, attitude and behaviour scores. Spearman correlation analysis was used to analyse the correlation between age and neurological work years and dysphagia nursing knowledge, belief and practice scores. P < 0.05 indicated a statistically significant difference.

## Results

### Single-factor analysis of the general information and its impact on dysphagia management knowledge scores among orthopaedic nurses

This study investigated a total of 894 orthopaedic nurses, including 852 females (95.3%) and 42 males (4.7%) (Because in China, most of the nursing work is done by women). The survey results showed that there was a statistically significant difference in the scores of orthopaedic nurses with the highest education level, whether they had received training on dysphagia, and whether they had actively inquired about dysphagia-related knowledge (P < 0.05) (Table 1). The value in bold represents P < 0.05.

**Table 1:** Single-factor analysis results of swallowing disorder management knowledge (n = 894)

Demographic characteristic	Number (n)	Constituent ratio (%)	Knowledge	*F/t*	*p*
Age				0.882	0.450
≤ 29	240	26.8	16.3 ± 3.553		
30–39	413	46.2	16.3 ± 3.579		
40–49	180	20.1	15.8 ± 3.972		
≥ 50	61	6.9	16.1 ± 3.361		
Sex				0.597	0.551
Female	852	95.3	16.1 ± 3.669		
Male	42	4.7	16.5 ± 3.006		
Academic qualification				5.818	**0.003**
Diploma	131	14.7	16.0 ± 3.199		
Bachelor’s degree	726	81.2	16.1 ± 3.681		
Master’s or doctoral degree	37	4.1	18.1 ± 3.831		
Duration of working in orthopedics				1.015	0.339
< 3	174	19.5	16.4 ± 3.403		
3–5	103	11.5	16.5 ± 3.006		
6–10	196	22.0	16.2 ± 3.665		
11–20	302	33.8	16.0 ± 3.833		
> 20	119	13.2	15.8 ± 3.920		
Professional title				0.277	0.758
Junior	319	35.7	16.3 ± 3.389		
Intermediate	411	46.0	16.1 ± 3.690		
Advanced	164	18.3	16.1 ± 3.986		
Orthopaedic Specialist Nursing Qualification Certification				0.616	0.433
Yes	238	26.2	15.9 ± 3.868		
No	656	73.4	16.2 ± 3.552		
Participated in training related to dysphagia				3.089	**0.013**
Yes	24	2.7	17.9 ± 5.034		
No	870	97.3	16.1 ± 3.585		
Searching for information about dysphagia on the internet				1.069	**0.005**
Yes	459	51.3	16.5 ± 3.469		
No	435	48.7	15.7 ± 3.786		

### Scores for dysphagia management knowledge among orthopaedic nurses

The score for swallowing disorder management knowledge was 16.1 ± 3.640 (Table 2), with the following items having the greatest accuracy: “Aspiration pneumonia”, “Feeling food stuck in the throat”, “Postoperative swallowing disorder can be treated using specific tools to deliver food into the mouth”, and “Coughing while eating”. The lowest accuracy rate was for, “Avoid the consumption of viscous foods”, “Digestive dysfunction”. “The pressure of the patient’s airbag sleeve during surgery should be controlled above 50 cm H_2_O”. and “If the patient accidentally inhales, they will always cough”. The 8 items with the lowest and highest accuracy are shown in Table 3.

**Table 2:** Current status of knowledge, attitudes, and behaviour scores among orthopaedic nurses

Items	Number of entries	Total score	Actual Score
Knowledge	30	30	16.1 ± 3.640
Attitude	8	40	32.5 ± 4.623
Behaviour	12	60	43.6 ± 11.513

**Table 3:** The items with the highest and lowest scores in dysphagia management knowledge

Items	Correct answer (%)
**The 5 items with the lowest accuracy rate**	
Avoid the consumption of viscous foods	8.5
If the patient accidentally inhales, they will always cough	9.1
Digestive dysfunction	9.6
The pressure of the patient’s airbag sleeve during surgery should be controlled above 50 cm H_2_O	11.3
**The 5 items with the highest accuracy rate**	
Aspiration pneumonia	90.2
Coughing while eating	88.6
Feeling food stuck in the throat	87.2
Postoperative use of specific instruments to deliver food into the mouth	82.7

### Attitude and behaviour scores of orthopaedic nurses for dysphagia management

The survey showed that 894 orthopaedic nurses had a nursing attitude score of 32.5 ± 4.623 (Table 2) regarding swallowing disorders in patients after cervical spine surgery. The top two nurses with the highest scores and the bottom two nurses with the lowest scores are shown in Table 4. The nursing behaviour score of 894 orthopaedic nurses for swallowing disorders in patients after cervical spine surgery was 43.6 ± 11.513 points (Table 2). The top 3 with the highest scores and the bottom 3 with the lowest scores are shown in Table 5.

**Table 4:** The items with the highest and lowest scores in dysphagia management attitudes

Items	Average score of items
**The 2 items with the lowest scores**	
Do you agree that swallowing disorder screening is the responsibility of nurses?	3.48 ± 1.088
Do you agree that swallowing disorder screening is the responsibility of a rehabilitation therapist?	3.81 ± 0.940
**The 2 items with the highest scores**	
Do you agree to inform other medical staff when a patient has swallowing disorders?	4.44 ± 0.614
Do you agree that all patients undergoing cervical spine surgery should undergo swallowing function screening?	4.27 ± 0.676

**Table 5:** The items with the highest and lowest scores in dysphagia management behaviours

Items	Average score of items
**The 3 items with the lowest scores**	
Record the screening results of swallowing function in patients undergoing anterior cervical surgery.	3.11 ± 1.387
Perform swallowing function screening for each patient undergoing cervical spine surgery.	3.28 ± 1.286
After cervical spine surgery, patients with swallowing disorders are screened daily to identify patients who quickly recover swallowing function.	3.34 ± 1.305
**The 3 items with the highest scores**	
Guiding or assisting patients with swallowing disorders in selecting appropriate positions and postures.	3.99 ± 1.025
Guiding patients with swallowing disorders during cervical spine surgery to maintain good oral hygiene to reduce the incidence of associated pneumonia.	3.88 ± 1.104
If there is a risk of swallowing oral medication, consult pharmacists and physicians for advice.	3.87 ± 1.104

### Correlation analysis of nursing knowledge, attitudes, and behaviours of orthopaedic nurses and dysphagia after anterior cervical spine surgery

The survey results showed that there was a positive correlation between the knowledge, attitude, and behaviour scores of orthopaedic nurses for dysphagia after anterior cervical surgery (P < 0.01) (Table 6).

**Table 6:** The correlation among knowledge, attitude, and behaviour scores of orthopaedic nurses

Items	knowledge	Attitude	Behaviour
knowledge	1		
Attitude	0.167**	1	
Behaviour	0.178**	0.467**	1

^**p < 0.01^

## Discussion

The results of this study showed that orthopaedic nurses had a knowledge score of 16.1 ± 3.640 for swallowing disorders after anterior cervical spine surgery, which was a moderate score compared to a full score of 30. The knowledge level needs to be improved. As shown by the survey data, orthopaedic nurses have a good understanding of typical symptoms and complications of swallowing disorders, such as “Coughing while eating”, “Feeling food stuck in the throat” and “Aspiration pneumonia”. However, they lack knowledge of implicit aspiration. The correct rate of the item “If the patient accidentally inhales, they will always cough” was relatively low (9.1%), indicating that 90.9% of orthopaedic nurses could not recognize the occurrence of silent aspiration, which is consistent with findings in other studies [11–12]. Silent aspiration refers to the infiltration of food, liquid, or saliva under the glottis without causing coughing [13]. If the cough reflex is impaired, the risk of aspiration pneumonia is higher, with 15.6% of aspiration pneumonia reported to be caused by latent aspiration [14]. Pneumonia caused by silent aspiration has a higher mortality rate within one month than pneumonia caused by non implicit aspiration, so nurses need to recognize that silent aspiration poses a fatal risk to patients [15].

From the attitude of nurses towards the management of swallowing disorders after anterior cervical spine surgery, it was not clear who should be responsible for evaluation and screening, so the scores of the two related options were relatively low: “Do you agree that swallowing disorder screening is the responsibility of nurses?” (3.48 ± 1.088) and “Do you agree that swallowing disorder screening is the responsibility of a rehabilitation therapist?” (3.81 ± 0.940). However, nurses also believed patients should be evaluated for swallowing disorders, and this score was very high (4.27 ± 0.676).

Therefore, managers need to develop standardized management models, clarify responsibilities, implement targeted training for orthopaedic nursing staff, and carry out practical operations from aspects such as screening and evaluation of dysphagia, rehabilitation nursing techniques for swallowing disorder patients, health education for swallowing disorder patients, and how to carry out quality control. This will enable nursing staff to master the basic theoretical knowledge and operational techniques of dysphagia and realize the importance of dysphagia assessment, and through continuous supervision by specialized teams, the knowledge and operation of nurses can be continuously improved [16]. In addition, it is necessary to implement multidisciplinary team collaboration to enable team members to clarify their job responsibilities, and each team member should manage patients from all aspects, such as swallowing assessment [17], rehabilitation training, and dietary management. Questions should be discussed, and the patient’s eating guidance and swallowing function training methods should be adjusted in a timely manner to improve the patient’s swallowing function, ensure nutrient intake, and ensure safe eating. Emphasizing the participation of multidisciplinary teams in practice can provide a more comprehensive and multidimensional approach to managing dysphagia in patients, improving the professionalism and efficiency of dysphagia management [18–19].

This study found that the accuracy rate of “avoiding the consumption of viscous liquids” and “eating liquid foods is safest” is low, indicating that most of nurses have incorrect understanding of the diet of elderly people with swallowing disorders. Choosing the correct food intake is the primary task of swallowing disorder management [20]. A study [21] found that attention should be given to nutrient intake after orthopaedic spine surgery, especially cervical spine surgery. Adequate nutritional consumption is essential for addressing the surgical stress response and mitigating the loss of muscle mass, strength, and functionality. However, inappropriate food can also lead to aspiration. Improving the safety and effectiveness of swallowing by adjusting the texture of patients’ food and drinks is currently a commonly used form of intervention for swallowing disorders [22]. Therefore, managers should develop a dietary plan after anterior cervical surgery to reduce aspiration rates and avoid malnutrition in patients.

The correlation analysis of the knowledge, attitudes, and behaviours of orthopaedic nurses in managing swallowing disorders after anterior cervical surgery showed a positive correlation (*P* < 0.01). At present, orthopaedic nurses had a moderate score in their knowledge of swallowing disorders after anterior cervical spine surgery, and their knowledge level needs to be improved. Due to a lack of relevant knowledge and skills, nursing attitudes are often not proactive enough, which can affect corresponding behaviors [23]. This can be achieved through support and education systems aimed at adjusting and improving the knowledge structure of nurses [24]. Therefore, managers can try to focus on providing knowledge and skills training for orthopaedic nurses on swallowing disorder nursing, aiming to improve their nursing knowledge level for postoperative swallowing disorders, thereby improving their attitudes and behaviours and ultimately improving patient outcomes [25].

## Study limitations

This study has the following limitations: the swallowing disorders management knowledge questionnaire developed by Rhoda was first applied in hospitals for patients with dysphagia after anterior cervical spine surgery in orthopaedics, and its reliability and validity need to be further verified; the research scope was relatively small, and only some hospitals in Chongqing were surveyed. The research conclusions still need to be further confirmed through multicentre and large-sample studies.

## Conclusions

In summary, this study investigated the current status of knowledge, attitudes, and behaviours of orthopaedic nurses in multiple hospitals regarding swallowing disorders after anterior cervical spine surgery. The results showed that nurses had more positive nursing attitudes, with moderate levels of swallowing disorder knowledge and nursing behaviours, which need to be improved, especially in areas such as implicit aspiration recognition and dietary choices. Nursing education institutions and hospitals should increase training related to swallowing disorders and encourage nurses to continue education and improve their knowledge, belief, and practice levels regarding swallowing disorders.

### Electronic supplementary material

Below is the link to the electronic supplementary material.

**Supplementary Material 1:** Questionnaire on knowledge, attitude, and behavior of dysphagia after anterior cervical spine surgery

## Data Availability

The datasets during and/or analysed during the current study available from the corresponding author on reasonable request.
