# Supplementary material for: A multicenter cross-sectional survey of the knowledge, attitude, and behaviour of nurses regarding dysphagia after anterior cervical spine surgery: a prospective multicentre study
Source: BMC Nurs. 2024 Jan 29;23:74. doi: 10.1186/s12912-023-01690-2 (PMC10823728; doi:10.1186/s12912-023-01690-2)
Supplement: Supplementary file 1 — Supplementary Material 1: Questionnaire on knowledge, attitude, and behavior of dysphagia after anterior cervical spine surgery [file 12912_2023_1690_MOESM1_ESM.pdf]

Knowledge of dysphagia

1. Do you agree with the following description of symptoms of dysphagia?

Variable	Disagree	Unable to decide	Agree	Score
Coughing while eating			※	1
skin allergies	※			1
Feeling food stuck in the throat			※	1
Less oral secretions	※			1
Food residue in the mouth			※	1
Insufficient chewing			※	1
If the patient accidentally inhales, they will always cough	※			1
Food reflux after eating			※	1
Frequent throat clearing after swallowing			※	1
Hoarse voice			※	1
Pain during swallowing			※	1
A burning sensation in the throat			※	1
**Total score**	–	–	–	12

2.Do you agree with the following description of complications related to dysphagia?

Variable	Disagree	Unable to decide	Agree	Score
Aspiration pneumonia			※	1
Anaphylactic shock	※			1
General weakness			※	1
Digestive dysfunction	※			1
Dehydration			※	1
Heart Attack	※			1
Haematemesis	※			1
Weight loss			※	1
Sleep disorders			※	1
**Total score**	–	–	–	9

2. Do you agree with the following description regarding the management of dysphagia?

Variable	Disagree	Unable to decide	Agree	Score
Patients need daily oral hygiene (gargling and brushing teeth) after surgery			※	1
Avoid the consumption of viscous foods.	※			1
The pressure of the patient’s airbag sleeve during surgery should be controlled above 50 cm H2O	※			
Eating water like liquids is the safest option	※			1
Local use of steroids during surgery can exacerbate the occurrence of postoperative swallowing disorders	※			1
The use of intraoperative bone morphogenetic proteins can exacerbate the occurrence of postoperative swallowing disorders			※	
The best posture for feeding a patient is to lie flat	※			1
Postoperative use of specific instruments to deliver food into the mouth			※	1
Neck auscultation can serve as an independent tool for diagnosing postoperative swallowing disorders	※			1
**Total score**	−	−	−	9

Attitudes

1. Do you agree that all patients undergoing cervical spine surgery should undergo swallowing function screening?

2.Do you agree that patients after anterior cervical surgery should undergo swallowing function screening before eating or drinking?

3. Do you agree to inform other medical staff when a patient has dysphagia?

4. Do you agree to use drinking water testing as the most commonly used screening method in your work?

5. Do you agree to proficiently use a certain swallowing screening scale?

6. Do you agree that swallowing disorder screening is a doctor's responsibility?

7. Do you agree that swallowing disorder screening is the responsibility of nurses?

8. Do you agree that swallowing disorder screening is the responsibility of a rehabilitation therapist?

Behaviour

1. Perform swallowing function screening for each patient undergoing cervical spine surgery.
2. When the patient's condition changes or swallowing function changes, swallowing function should be reevaluated.

3. Guiding or assisting patients with swallowing disorders in selecting appropriate positions and postures.
4. Record the screening results of swallowing function in patients undergoing anterior cervical surgery.
5. After cervical spine surgery, patients with swallowing disorders are screened daily to identify patients who quickly recover swallowing function.
6. Guiding or assisting patients with swallowing disorders in selecting appropriate positions and postures.
7. Conduct nutritional risk screening for each postoperative cervical spine patient.
8. Teach patients and family members to identify clinical manifestations and complications of swallowing disorders.
9. If there is a risk of swallowing oral medication, consult pharmacists and physicians for advice.
10. Guide or assist patients with swallowing disorders in swallowing function rehabilitation.
11. Assist patients with swallowing disorders in selecting appropriate compensatory eating measures.
12. Assist patients in modifying the environment before eating, such as reducing interference, reducing noise, increasing lighting, etc.
